# Food insecurity in urban American Indian and Alaska Native populations during the COVID-19 pandemic

**DOI:** 10.1186/s12889-024-18390-4

**Published:** 2024-03-27

**Authors:** Katie Nelson, Alexandra M. Jackson, Cassandra J. Nguyen, Carolyn Noonan, Clemma Muller, Richard F. MacLehose, Spero M. Manson, Denise A. Dillard, Dedra Buchwald, CONCERTS Collaborative, Odile Madesclaire, Austin Henderson, Talia Quandelacey

**Affiliations:** 1 Institute for Research and Education to Advance Community Health, Washington State University, 1100 Olive Way, Ste 1200, Seattle, WA 98101 USA; 2 Pacific University, Forest Grove, OR USA; 3 Department of Nutrition, University of California, Davis, Davis, CA USA; 4 Division of Epidemiology and Community Health, Division of Environmental Health Sciences, University of Minnesota School of Public Health, Minneapolis, MN USA; 5 Colorado School of Public Health, Centers for American Indian and Alaska Native Health, University of Colorado Anschutz Medical Campus, Aurora, CO USA; 6 Denver Indian Health and Family Services, Southcentral Foundation, Indian Health Board of Minneapolis, First Nations Community Healthsource, Hunter Health Clinic, Albuquerque, USA

**Keywords:** Food insecurity, COVID-19, American Indian/Alaska Native, Health disparities

## Abstract

**Background:**

Food insecurity is an important social determinant of health that was exacerbated by the COVID-19 pandemic. Both food insecurity and COVID-19 infection disproportionately affect racial and ethnic minority groups, particularly American Indian and Alaska Native communities; however, there is little evidence as to whether food insecurity is associated with COVID-19 infection or COVID-19 preventive behaviors such as vaccination uptake. The purpose of this study was to evaluate associations between food insecurity, COVID-19 infection, and vaccination status among urban American Indian and Alaska Native adults seen at 5 clinics serving urban Native people.

**Methods:**

In partnership with health organizations in Alaska, Colorado, Kansas, Minnesota, and New Mexico, the study team conducted a cross-sectional survey in 2021 to assess food security status and attitudes, barriers, and facilitators for COVID-19 testing and vaccination. Logistic regression was used to examine the association of food security status with sociodemographic factors and COVID-19 infection and vaccination status. Marginal standardization was applied to present results as prevalence differences.

**Results:**

Among 730 American Indian and Alaska Native adults, the prevalence of food insecurity measured during the pandemic was 38%. For participants who reported persistent food security status before and during the pandemic (*n* = 588), the prevalence of food insecurity was 25%. Prevalence of COVID-19 infection and vaccination did not vary by food security status after adjustment for confounders.

**Conclusions:**

High rates of food insecurity among American Indian and Alaska Native communities likely increased during the COVID-19 pandemic. However, despite the high prevalence of food insecurity, community-led efforts to reduce COVID-19 infection and increase vaccination uptake across Indian Health Service and Tribal healthcare facilities may have mitigated the negative impacts of the pandemic for families experiencing food insecurity. These successful approaches serve as an important reference for future public health efforts that require innovative strategies to improve overall health in American Indian and Alaska Native communities.

## Introduction

American Indian and Alaska Native (AIAN) people are disproportionately impacted by many social determinants of health associated with increased COVID-19 infection prevalence and severe morbidity and mortality [1–3]. Food insecurity, in particular, is a complex and important social determinant of health that affects AIAN communities at significantly higher rates than other racial and ethnic groups [4]. The forced relocation of AIAN communities from traditional lands disrupted their way of life and access to traditional healthy foods. These historical forces contribute to modern AIAN experiences, including inequitable food access, high prevalence of food insecurity, and disparate burden of diet-related health outcomes [4–7]. Food insecurity is defined as having unreliable or limited access to affordable, nutritious food and is associated with higher risk of physical and mental health conditions, including type 2 diabetes, hypertension, chronic stress, anxiety, and depression [8, 9]. AIAN people experience disparities in these health conditions, which also confer a high risk of severe complications, hospitalization, and death following COVID-19 infection [10, 11]. Despite these challenges, Tribal entities were quick to design and implement COVID-19 vaccination campaigns, which contributed to high rates of vaccination in AIAN communities early in the pandemic [12].

Prior to the COVID-19 pandemic, AIAN communities across the U.S. experienced a higher prevalence of food insecurity (27%) than African American (21%), Asian American and Pacific Islander (6%), Hispanic (17%), and non-Hispanic White populations (9%) [13]. However, during the COVID-19 pandemic, food insecurity initially sharply increased throughout the U.S. due to job or income loss, school closures, social isolation, and food supply chain disruptions [14]. Food insecure families living in poverty may be at a greater risk of COVID-19 infection due to increased exposure from work obligations outside the home (particularly for essential workers), more frequent use of public transportation, more interactions with other individuals who are vulnerable due to increased exposure, and lack of healthcare access [15–17]. In addition, food insecurity may be associated with malnutrition, nutrient deficiencies, and a higher risk of chronic disease, which may increase susceptibility to COVID-19 [18]. Food insecurity during the pandemic was also associated with a high likelihood of COVID-19 infection in older U.S. adults and in counties with large African American or large American Indian or Alaska Native populations [16, 19]. One study in an Iranian population found that after controlling for confounders, food insecurity increased the risk of COVID-19 threefold [20]. Data from the 2021 National Health Interview Survey revealed that food insecurity was independently associated with COVID-19 after controlling for sociodemographic and health-related factors [21]. Food insecurity may also negatively impact vaccination uptake [22]. Research shows that individuals experiencing food insecurity may be less likely to engage in preventative health behaviors such as vaccinations due to cost (or perceived costs) and vaccine hesitancy, distrust in government, and logistical barriers [23, 24]. Little research has explored food insecurity among AIAN people during the pandemic, likely in part due to the unique circumstances of research partnerships with AIAN communities during the pandemic, despite their high risk of food insecurity [25–28]. Likewise, individual-level associations among food insecurity, COVID-19 infection, and COVID-19 vaccination have not been examined in any AIAN population.

We conducted a cross-sectional survey in collaboration with 5 geographically dispersed health organizations that serve urban AIAN populations to examine the association between sociodemographic factors and food insecurity. We also evaluated the association between food insecurity and COVID-19 infection, and food insecurity and vaccination uptake mid-pandemic. We hypothesized that food insecurity would be a risk factor for COVID-19 infection and associated with lower rates of vaccination, after adjusting for potential confounding by sociodemographic, cultural, and health-related factors. As an exploratory analysis, we examined the association between persistent food security and COVID-19 infection and vaccination, which is an important area of inquiry within the COVID-19 context where there were newly food insecure households [29, 30].

## Methods

### Study setting

Community Organizations for Natives: COVID-19 Epidemiology, Research, Testing, and Services (CONCERTS) was designed to identify and remove barriers to COVID-19 testing and vaccination among urban AIAN adults. In partnership with 5 geographically dispersed health organizations serving primarily AIAN people in urban settings, the CONCERTS study team created and implemented a cross-sectional survey to assess attitudes, barriers, facilitators, and risk factors for COVID-19 testing and vaccination. The survey was developed using the National Institutes of Health RADx-UP Common Data Elements and PhenX Toolkit with feedback from each site to ensure the appropriateness and relevance of survey questions [31]. The urban areas served by the participating sites include Albuquerque, NM, Anchorage, AK, Denver, CO, Minneapolis-St. Paul, MN, and Wichita, KS. Surveys were administered between January 2021 and May 2021.

Eligibility for the study included being a patient at any of the 5 primary care clinics within the prior year, ≥ 18 years of age, identified as American Indian or Alaska Native, and not diagnosed with dementia or other serious cognitive issues (ICD-10 codes F01-04, G30, or G31) in the Electronic Health Record system (EHR). Using the EHR, we stratified sampling by age (18–54 vs. 55 and over) and sent eligible clinic patients an email invitation to participate in the survey. Patients without an email address were mailed invitations. Potential participants received up to 4 reminders over a 14-day period. Our goal was to enroll up to 150 participants per clinic, based on sample size calculations for the examination of barriers to COVID-19 testing. If this was not achieved after the first round of recruitment, we selected a new random sample of eligible clinic patients. The survey took approximately 40 min to complete, and participants received a $100 gift card as compensation. This study was approved by the Washington State University IRB (#18590), the Alaska Area Institutional Review Board (#2020-11-044), Tribal research review committees, and the Alaska Native Tribal Health Consortium. Tribal approval was also obtained for dissemination.

### Survey measures

#### Food insecurity

Food security status was assessed using the U.S. Department of Agriculture’s 6-item short form of the Food Security Survey Module, shown in Table 1 [32]. This survey has been shown to identify households experiencing food insecurity with reasonably high specificity and sensitivity and minimal bias compared with the 18-item USDA measure, and is less burdensome for respondents. The number of affirmative responses was summed and scores of 0–1 were coded as food secure whereas scores of 2–6 were coded as food insecure, in alignment with the published scoring procedures.

**Table 1:** USDA 6-item short form food security survey questions

Questions	Answer Options
In the **past 12 months**, the food that my household bought just didn’t last, and there wasn’t enough money to get more	Often true; Sometimes true; Never true
In the **past 12 months**, my household couldn’t afford to eat balanced (healthy) meals.	Often true; Sometimes true; Never true
In the **past 12 months**, did you or anyone in your household ever cut down the size of your meals or skip meals because there wasn’t enough money for food?	Yes; No
In the **past 12 months**, how often did this happen?	Almost every month; Some months but not every month; Only 1 or 2 months
In the **past 12 months**, did you ever eat less than you felt you should because there wasn’t enough money to buy food?	Yes; No
In the **past 12 months**, were you ever hungry but didn’t eat because you couldn’t afford enough food?	Yes; No

We created an additional variable hereafter called “persistent” food security status by combining income change and food security status during the pandemic. Income change was determined using the question: “Since the beginning of the COVID-19 pandemic in February 2020, has your income…” with response categories of stayed the same, increased, or decreased. Persistent food security status was considered *insecure* if a participant was classified as food insecure during the pandemic (using the Food Security Survey Module described above) and reported either the same or increased income since the onset of the pandemic. Persistent food security status was considered *secure* if a participant was classified as food secure during the pandemic and reported either the same or decreased income since the onset of the pandemic. Participants who were classified as food insecure during the pandemic and reported decreased income since the onset of the pandemic (*n* = 127) and participants who were classified as food secure during the pandemic and reported the same or increased income since the onset of the pandemic (*n* = 65) had persistent food security status set to missing since their status may have changed during the pandemic. Participants with persistent food *insecurity* were assumed to have been food insecure both before and during the pandemic while participants with persistent food *security* were assumed to have been food secure both before and during the pandemic.

#### COVID-19 infection

COVID-19 infection was determined using two questions: (1) “Has a doctor, nurse or other health care professional ever told you that you were infected with or have COVID-19?” and (2) “Did any test indicate that you had COVID-19?” A participant was considered to have had a COVID-19 infection if they responded yes to either question.

#### COVID-19 vaccination

COVID-19 vaccination status was assessed by asking “Have you received a COVID-19 vaccine?” Response categories included yes or no.

#### Sociodemographic factors

Sociodemographic characteristics included in the analysis were self-reported age, sex assigned at birth, current marital status, level of completed education, current employment status, and household income in the past 12 months.

### Statistical analysis

We first examined the association between sociodemographic factors and food security status using logistic regression. Separate models were fit for each sociodemographic factor as the only independent variable in the model and food security status as the dependent variable. Although age was measured as a continuous variable, it was included in the model as a categorical variable (18–35, 36–54, 55–64, 65 + years) for ease of interpretation. Next, we estimated the association of food security status (independent variable) with self-reported COVID-19 infection and vaccination status (dependent variables) using separate logistic regression models for each outcome. For each association, we fit an unadjusted model and a model adjusted for health organization (as a categorical variable), sex at birth (as a categorical variable), age (as a continuous variable), education (as a categorical variable), and marital status (as a categorical variable). Marginal standardization was used to calculate the predicted prevalence of each outcome by food security status and the prevalence difference according to food security status [33, 34]. These results are absolute effects and interpreted as the average difference in the prevalence of the outcome that would be expected comparing a group of individuals experiencing food insecurity to individuals not experiencing food insecurity, adjusted for the distribution of confounders as in the study sample.

Exploratory analyses were restricted to participants for whom we could estimate persistent food security status. We used the logistic models to evaluate associations between persistent food insecurity (vs. persistent food security) and COVID-19 infection and vaccination. All analyses incorporated inverse probability weights to account for age-based sample selection and nonresponse according to age and sex. Weights were scaled so that each of the five participating sites contributed equally to the final analyses. All analyses were conducted in STATA and included participants with complete-case data [35].

## Results

Of 4,603 eligible clinic patients contacted, 788 (17%) were enrolled and completed the survey. Of these, 730 had complete data to assess food insecurity and were included in the analyses.

### Prevalence of food insecurity

The prevalence of food insecurity across all health organizations was 38%. Table 2 presents the distribution of sociodemographic factors and the prevalence of food insecurity by sociodemographic factors. The prevalence of food insecurity was lower among participants aged 55 years and older compared to those aged 18 to 35 years. Food insecurity was 19% less prevalent among individuals with a college degree compared to those with less than a high school degree (95% CI: -32%, -6%). The prevalence of food insecurity was also lower among those with higher compared to lower income. Conversely, the prevalence of food insecurity was 11% higher among females compared to males (95% CI: 2%, 20%) and 18% higher among individuals who were divorced, separated, or widowed compared to those who were married or a member of an unmarried couple (95% CI: 7%, 30%). Similarly, individuals who were unemployed and disabled had 20% (95% CI: 5%, 35%) and 26% (95% CI: 9%, 44%) higher prevalence of food insecurity, respectively, than their counterparts who were employed.

**Table 2:** Prevalence of food insecurity by sociodemographic factors among 730 urban American Indian and Alaska Native survey participants, January-May 2021^a^

Factor	%	% Food Insecurity	Food Insecurity PD (95% CI)
Age in years			
18–35	34%	39%	Reference
36–54	41%	41%	2% (-9%, 13%)
55–64	16%	29%	-10% (-21%, 0%)
65+	9%	35%	-4% (-18%, 9%)
Sex			
Male	40%	32%	Reference
Female	60%	42%	11% (2%, 20%)
Marital status^b^			
Married or member of an unmarried couple	42%	33%	Reference
Divorced, separated, or widowed	21%	51%	18% (7%, 30%)
Never married	37%	37%	5% (-6%, 15%)
Education^c^			
Less than high school, no diploma	8%	44%	-1% (-18%, 17%)
High school graduate or GED	23%	44%	Reference
Some college	29%	37%	-7% (-20%, 6%)
Associate, occupational, technical, or vocational degree	17%	45%	1% (-14%, 16%)
College degree or higher	24%	25%	-19% (-32%, -6%)
Current employment status^d^			
Employed full- or part-time	63%	32%	Reference
Unemployed	13%	52%	20% (5%, 35%)
Retired	7%	33%	1% (-12%, 13%)
Disabled	6%	59%	26% (9%, 44%)
Other	10%	42%	10% (-6%, 26%)
Total household income in past 12 months^e^			
<$15,000	23%	64%	Reference
$15,000-$34,999	27%	49%	-16% (-28%, -3%)
$35,000-$59,999	25%	28%	-36% (-49%, -23%)
$60,000+	24%	10%	-54% (-65%, -43%)

*PD* Prevalence difference: absolute effect showing prevalence of food insecurity for the indicated category compared to the reference category, *CI* Confidence interval

^a^ Unweighted sample size, results weighted for sampling and nonresponse

^b^*n* = 6 missing

^c^*n* = 5 missing

^d^*n* = 4 missing

^e^*n* = 12 missing

### Association of food insecurity with COVID-19 infection and vaccination status

The association of food insecurity with COVID-19 infection and vaccination status, unadjusted and adjusted for sociodemographic characteristics is shown in Table 3. The prevalence of COVID-19 infection was 4% higher among individuals classified as food insecure compared to those classified as food secure (95% CI: -3%, 12%), and adjustment for confounders had no impact on the magnitude of the difference (PD: 4%, 95% CI: -3%, 11%). Prevalence of COVID-19 vaccination was 11% lower among individuals classified as food insecure compared to those classified as food secure (95% CI: -21%, -2%); however, adjustment for confounders reduced the magnitude of the difference (PD= -2%; 95% CI: -10%, 6%).

**Table 3:** Association of food insecurity with COVID-19 infection and vaccination status among urban American Indian and Alaska Native survey participants, January-May 2021

	COVID-19 infection			COVID-19 vaccination		
	**% (N**^a^**)**	**Unadjusted** **PD (95% CI)**	**Adjusted**^b^ **PD (95% CI)**	**% (N**^a^**)**	**Unadjusted** **PD (95% CI)**	**Adjusted**^b^ **PD (95% CI)**
***Full cohort***	*N*^a^ *= 704*			*N*^a^ *= 690*		
Food secure	14% (60)	Reference		54% (303)	Reference	
Food insecure	18% (38)	4% (-3%, 12%)	4% (-3%, 11%)	43% (130)	-11% (-21%, -2%)	-2% (-10%, 6%)
***Persistent food security status***^***c***^	*N*^a^ *= 537*			*N*^a^ *= 529*		
Food secure	15% (57)	Reference		54% (271)	Reference	
Food insecure	14% (17)	-1% (-9%, 8%)	-1% (-9%, 7%)	44% (72)	-10% (-22%, 2%)	-2% (-13%, 9%)

*PD* Prevalence difference: absolute effect showing the prevalence of COVID-19 infection or vaccination among food insecure individuals compared to food secure individuals, *CI* Confidence interval

^a^Unweighted sample size, results weighted for sampling and nonresponse

^b^Adjusted for health organization clinic, sex, age, education, and marital status

^c^Restricted to the subset of respondents categorized as having persistent status (food insecure, food secure) before and during the pandemic

### Persistent food security

We included 558 participants who were classified as having persistent food security or insecurity status in an exploratory analysis to examine associations between food insecurity and COVID-19 testing and vaccination specifically for participants whose food security status likely did not change as a result of the pandemic. Among those with persistent food security status (i.e., those likely to have had the same food security status prior to and during the pandemic), the prevalence of food insecurity captured at the time of the study was 25%. Sociodemographic factors among those who had persistent food security status, stratified by food security status at the time of the survey, are presented in Table 4. The exploratory analysis showed similar associations between persistent food insecurity and COVID-19 infection and vaccination as the full cohort analysis (Table 3).

**Table 4:** Sociodemographic factors according to food security status among participants classified as having persistent food security status before and during the pandemic

	Food Secure	Food Insecure
	(*n* = 429)^a^	(*n* = 129)^a^
Age in years, *%*		
18–35	32%	36%
36–54	40%	43%
55–64	18%	11%
65+	10%	9%
Female, *%*	54%	63%
Marital status, *%*		
Married or member of an unmarried couple	46%	33%
Divorced, Separated, or widowed	17%	29%
Never married	37%	38%
Education, *%*		
Less than high school, no diploma	8%	9%
High school graduate or GED	22%	30%
Some college	29%	30%
Associate, occupational, technical, or vocational degree	15%	14%
College degree or higher	27%	17%
Current employment status, *%*		
Employed full- or part-time	67%	60%
Unemployed	10%	11%
Retired	8%	8%
Disabled	3%	14%
Other	11%	7%
Total household income in past 12 months, *%*		
<$15,000	14%	39%
$15,000-$34,999	25%	37%
$35,000-$59,999	28%	16%
$60,000+	33%	8%

^a^Unweighted sample size, results weighted for sampling and nonresponse

## Discussion

Food insecurity is an important social determinant of health and increased in many communities because of the pandemic. 38% of study participants experienced food insecurity during the pandemic (2021) without differences in COVID-19 infection or vaccination prevalence by food insecurity status after adjustment.

Observed associations between sociodemographic characteristics and reported food insecurity were similar to those found in previous studies conducted in the U.S. [36]. Our results add to the literature indicating an increase in food insecurity among urban AIAN populations since the onset of the COVID-19 pandemic [37, 38]. While slightly lower than the 50% food insecurity reported in the February 2021 survey conducted by the Native American Agriculture Fund, the experience of food insecurity was high in the study sample [39]. Notably, among participants whose food security status likely did not change due to the onset of the pandemic, only 25% of participants reported being food insecure, compared to 38% for the overall sample, suggesting a substantial increase since the onset of the pandemic.

A high prevalence of food insecurity among AIAN families has also been reported prior to the pandemic [40]. A recent scoping review of pre-pandemic food insecurity in AIAN people found a weighted average prevalence of food insecurity of 46%; however, studies varied widely with food insecurity ranging from 16 − 80% [4]. Regardless, food insecurity among AIAN communities is likely significantly higher than the nationwide estimate of 10.5% in 2019 [8]. Most research has focused on food insecurity in rural- and/or reservation-living AIAN households, which experience additional challenges to food access such as restricted transportation options and walkability, and reliable internet and/or phone services [4]. However, in studies that included urban and rural AIAN households, urban households were more likely to report food insecurity than their rural counterparts [40, 41]. Higher food insecurity rates in urban areas may be in part due to limited access to traditional foods or food obtained through subsistence activities, exclusion from reservation-based programs for AIAN families (such as the Food Distribution Program on Indian Reservations), and separation from community or family support [4]. Despite these challenges, many AIAN people have established strong communities within urban settings, and organizations such as Native American LifeLines have found innovative ways to provide access to culturally appropriate food and healthcare for urban AIAN families [42]. Additional research to elucidate the reasons for food insecurity and novel strategies to reduce food insecurity in both urban and rural areas are needed.

Research among adults in the U.S. indicated significant increases in food insecurity at the onset of the COVID-19 pandemic [43, 44]. However, thus far, research on food insecurity among AIAN communities during the COVID-19 pandemic has been limited, with two studies suggesting increases in food insecurity since the pandemic. One study conducted in the Blackfeet Indian Tribal Community found that 79% of survey respondents indicated some degree of food insecurity over a 4-month period in late 2020 [37, 38]. Similarly, few studies have examined associations between food insecurity and COVID-19 infection or vaccine uptake. However, our findings that food insecurity was not associated with COVID-19 infection or vaccination, differ from prior research among other populations. Prior studies in older adults and Latinx families suggest higher prevalence of COVID-19 infection in households experiencing food insecurity [16, 45]. However, in one of the studies, the relationship was no longer significant after adjusting for house size and number of residents [45].

While research in other communities across the U.S. indicates that people with food insecurity were less likely to receive COVID-19 vaccines, this was not evident in this sample of urban AIAN adults after adjusting for confounders [46, 47]. Many Tribal organizations responded swiftly to the COVID-19 pandemic and were early adopters of public health vaccination campaigns, with timely distribution of vaccines to Indian Health Service and Tribal healthcare facilities [12]. Federal data from April 2021 showed that more AIAN people (32%) were fully vaccinated against COVID-19 than Whites (19%), Asian Americans (16%), and African Americans (12%) [48]. Despite a high prevalence of food insecurity in this sample, the community-led vaccination campaigns across Indian Health Service and Tribal healthcare facilities may have mitigated the negative impacts of food insecurity on COVID-19 preventive behaviors such as vaccination [12]. However, the unadjusted results suggest that public health strategies that direct resources to food assistance programs may be useful in improving access to COVID-19 vaccinations for families experiencing food insecurity. For example, offering vaccinations at charitable food distribution sites, which faced challenges but were nimble and able to adapt in the face of the public health emergency [49]. Alternatively, efforts to screen for food security in healthcare settings could be used to simultaneously direct individuals towards food assistance as well as preventive health services [50]. As these findings differ from prior findings, additional research examining what protective factors and the potential mechanisms that may have occurred is an important next step.

The implementation and scaling up of secondary interventions that address food insecurity among urban AIAN communities is a growing priority. Two interventions have reduced food insecurity among AIAN children [51, 52]. One intervention with the Navajo Nation included a fruit and vegetable prescription and empowerment program, while the other intervention aimed to foster Indigenous culture and pride, including culturally-based gardening practices. The Indigenous Food Circle described an indigenous-led network to address the immediate needs of food insecurity exacerbated during the pandemic [53] that highlighted the importance of advocacy, Indigenous-led initiatives, and culturally appropriate frameworks to address the systemic issues of food insecurity in AIAN communities. A study conducted among Alaska Native women receiving the Special Supplemental Nutrition Program for Women, Infants, and Children (WIC) assistance showed that intake of traditional foods was associated with improved diet quality [54]. While an association between food security and traditional food intake was not observed, incorporating efforts to increase access to traditional foods and subsistence practices could strengthen programs and policies aimed at reducing health disparities experienced by AIAN communities.

These studies suggest multiple strategies may reduce food insecurity among Native communities. As the food environment and federal financial interventions (e.g., stimulus checks and child tax credit) are discontinued, understanding the impacts on food insecurity status is integral to reducing food insecurity among AIAN people. Working directly with AIAN populations to contextualize and create standardized measurements of food insecurity among rural and urban AIAN communities is critical to creating appropriate strategies to combat food insecurity and its negative impacts on health [4]. The U.S. Department of Agriculture food insecurity screening questions focus on the ability to *purchase* food, rather than *provide* food, and do not ask about subsistence farming or gardening, hunting, or fishing, which are common in many Native communities, though less so in urban areas [55–57]. Furthermore, it is imperative to recognize the importance of food sovereignty within AIAN communities and support ongoing efforts to address inequities within the food system [58].

Limitations of the present study include a cross-sectional design, which prevents our ability to establish causation, temporality, and proximity between food insecurity and COVID-19 infection or vaccination. Surveys were only sent to individuals who visited the clinic in the year prior to the pandemic, so our sample may have included more people who seek out consistent healthcare. Participants were able to complete the study on paper or online in an attempt to reach the community; however, it is possible that demographic groups may have been missed with this study design. Further, our response rate was low, raising the possibility of unknown selection bias. The study team did not ask participants to differentiate between laboratory-confirmed COVID-19 diagnosis and at-home COVID-19 tests, the latter yielding less accurate results. Additionally, we used the 6-item screener, which may not be a valid measurement of food insecurity for AIAN adults. As experiencing food insecurity likely changed for many households during the pandemic, we developed a variable combining income change and food security as a proxy measure to assess change. While exploratory, this proxy measure may not have accurately assessed change, and additional research exploring food insecurity as a fluctuating (and less stable) variable is necessary. Lastly, we collected data from January to May 2021, about one year after the onset of the pandemic. Access to vaccines varied across the partner sites, and we were not able to distinguish whether participants were not vaccinated due to access or availability. However, our study included a large sample of AIAN adults across the age range from geographically diverse areas, making it the largest and most comprehensive study to date of food insecurity among urban AIAN communities.

## Conclusions

This study sheds light on the pervasive issue of food insecurity among AIAN communities during the COVID-19 pandemic. Despite the high prevalence of food insecurity among the study participants, our findings show that food insecurity was not significantly associated with an increased risk of COVID-19 infection or decreased vaccination uptake. While the community-led efforts to alleviate the impacts of the pandemic appear to have mitigated the impact of food insecurity on COVID-19 preventive behaviors, the unadjusted results suggest that additional public health strategies may also be beneficial for preventing COVID-19 infection among those experiencing food insecurity. Addressing the systemic issues of food insecurity in AIAN communities will require a multifaceted approach that incorporates culturally appropriate initiatives and measures, as well as efforts to increase access to traditional foods.

## Data Availability

The datasets generated and analyzed during the current study are not publicly available in order to protect participant privacy. Contact Katie Nelson (katie.nelson@wsu.edu) with questions regarding data.

## Abbreviations

AIAN: American Indian/Alaska Native
CONCERTS: Community Organizations for Natives:COVID-19 Epidemiology, Research, Testing, and Services
EHR: Electronic Health Record(s)
USDA: United States Department of Agriculture
